# Development of a new wealth index for South Sudan: association between household wealth and malaria prevention practices in the context of seasonal malaria chemoprevention in Northern Bahr el Ghazal, South Sudan

**DOI:** 10.1186/s40249-025-01327-3

**Published:** 2025-07-01

**Authors:** Sikai Huang, Jamshed Khan, Francis Lokang, Abubaker Rom Ayuiel, Kevin Baker, Ahmed Julla, Sol Richardson

**Affiliations:** 1 Vanke School of Public Health, Tsinghua University, Beijing, China; 2 Malaria Consortium South Sudan, Plot 23, Block Government Contentment Area, Second Class Residential, Airport Road, Goshen House, Juba, South Sudan; 3 Malaria Consortium London, The Green House, 244-254 Cambridge Heath Road, London, E2 9DA UK; 4 Department of Global Public Health, Karolinska Institute, Stockholm, Sweden; 5 Department of National Malaria Control Program, Ministry of Health, Republic of South Sudan, HQs Ministerial Complex, P.O. Box 88, Juba, South Sudan

**Keywords:** Seasonal malaria chemoprevention, South Sudan, Household wealth, Child health

## Abstract

**Background:**

The World Health Organization recommends seasonal malaria chemoprevention (SMC) using sulfadoxine-pyrimethamine and amodiaquine (SPAQ) to prevent malaria among children aged 3–59 months in regions with marked seasonality of malaria transmission. Socioeconomic disparities in household malaria prevention within the SMC context remain uncharacterized. This study aimed to construct a household wealth index and examine its association with SMC implementation, children malaria infection, and malaria prevention practices in South Sudan.

**Methods:**

We utilized data from repeated cross-sectional household surveys conducted in Aweil County in 2022, involving 2767 households. The survey included asset-based questions tailored to the local context. We constructed a 12-item wealth score scale based on asset ownership using Mokken scale analysis and calculated weighted scores using multiple correspondence analysis to obtain wealth index quintiles. Survey-weighted logistic regressions were performed to assess the association of household wealth index quintiles with SMC implementation, children malaria infection, and malaria prevention practices.

**Results:**

The constructed 12-item wealth scale demonstrated strong internal consistency (Cronbach’s alpha = 0.72). However, households in the lower wealth quintiles (1st quintile) had lower odds of ownership of mosquito nets compared with those in the 3rd quintile [odds ratio (*OR*) = 0.12, 95% confidence interval (*CI*): 0.05–0.26,* P* < 0.001)]. Households in the highest wealth quintile (5th quintile) had higher odds of access to alternative malaria prevention tools (e.g., repellents) compared with the 3rd quintile (*OR* = 2.75, 95% *CI:* 1.30–5.83, *P* = 0.010). However, household wealth was not significantly associated with SMC implementation (household visits by SMC *boma* distributors, child receipt of Day 1 SPAQ, and caregiver SMC knowledge) or malaria infection outcomes within SMC context.

**Conclusions:**

The new wealth index tailored to South Sudan is a useful tool for assessing socioeconomic health determinants. While household access to SMC showed a low degree of wealth-associated disparities, reflecting the equitable coverage of the door-to-door SMC delivery model, significant inequities remain in household access to other malaria prevention practices, such as mosquito nets. These findings imply the need for strategies to enhance equity in distributing essential malaria prevention resources.

**Graphical Abstract:**

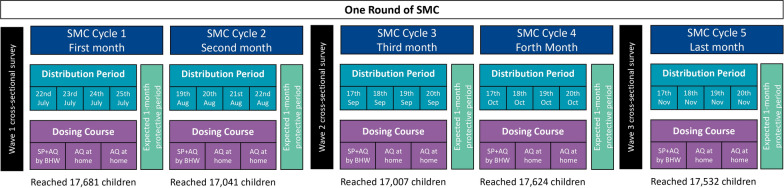

**Supplementary Information:**

The online version contains supplementary material available at 10.1186/s40249-025-01327-3.

## Background

Seasonal malaria chemoprevention (SMC) with intermittent administration of sulfoxide-pyrimethamine (SP) and amodiaquine (AQ) has been recommended by the World Health Organization (WHO) since 2012 [1]. This intervention is considered highly cost-effective, with a protective effectiveness of 88.2% in preventing clinical malaria in children under five during high-transmission periods [2]; previous work found that incremental economic cost per malaria case averted ranged from United States dollars (USD) 2.91 in Niger to USD 30.73 in the Gambia [3]. Since its recommendation, SMC has been implemented in 19 countries in the Sahel and other seasonal areas of Africa, reaching 53 million children per monthly cycle by 2023 [4]. In 2022, the WHO updated its guidelines on SMC, providing greater flexibility in terms of geography and age eligibility for malaria-endemic countries to scale up SMC [5, 6].

Malaria is endemic across South Sudan, accounting for approximately 66.8% of outpatient visits, 30% of hospital admissions, and 50% of all causes of death in the hospitals in 2019 [7, 8]. Similar to other Sahel countries, malaria transmission in South Sudan is highly seasonal, with a sharp increase in malaria cases during the rainy season from July to November and children under five years of age being the most vulnerable group [9]. However, ongoing conflict and insecurity, inadequate infrastructure, and adverse climatic events have significantly impeded malaria control efforts and hindered progress towards the milestones outlined in the *Global Technical Strategy for Malaria 2016–2030* [4, 10, 11].

In response to this burden, *Médecins Sans Frontières (MSF),* in collaboration with South Sudan’s Ministry of Health, piloted SMC in Yambio County, Western Equatoria State in 2019. This achieved coverage of 91.1% among children under five and demonstrated a 77.1% reduction in severe malaria incidence [12]. Building on this success, SMC has been included in South Sudan’s National Malaria Strategic Plan 2020–2025 [8]. SMC was implemented at scale for the first time in Aweil South County, Northern Bahr el Ghazal State, where approximately 18,000 children received at least one dose of SMC. A subsequent non-randomized controlled study for impact investigation reported an 82% lower odds of caregiver-reported rapid diagnostic test (RDT)-confirmed malaria episodes among children receiving SMC in Aweil South County [9]. 

While studies have explored ways to optimize SMC implementation in terms of delivery methods (e.g., door-to-door versus fixed point), adherence to antimalarial regimens and community-driven behavior change approaches [2, 3, 13–18], limited attention has been given to the role of household socioeconomic position (SEP) in shaping malaria prevention outcomes under programmatic conditions.

Almost 90% of all malaria-related morbidity and mortality occur in the world’s poorest regions [4]. South Sudan remains the poorest country in the world with a Gross Domestic Product of USD 492 (purchasing power parity; international dollars per capita, 2024) according to the International Monetary Fund [19]. Additionally, South Sudan’s Human Development Index value was 0.385, with 7 in 10 individuals living below the international poverty line, according to South Sudan Office Annual Report 2023 [20]. Understanding the SEP of target populations is critical for malaria risk stratification and tailoring health service delivery to improve intervention effectiveness [21].

Household SEP, encompassing indicators such as household income, asset ownership, housing quality, geographic location, and educational level, influences access to SMC and other malaria prevention tools. For example, an urban–rural comparative cross-sectional study across nine SMC-states of Nigeria reported a lower coverage of SMC and odds of SMC awareness, knowledge and belief in effectiveness among caregivers of eligible children in urban areas, despite their relatively higher literacy and level of education [22]. Asset indices are widely preferred as a measure of SEP in low-income countries, where stable income sources and regular consumption patterns are often unavailable [23]. Another study in Nigeria, utilizing an asset-based measure of SEP, found that wealthier households were more likely to access SMC through unofficial delivery channels, such as private purchase and unofficial fixed-point distribution [17]. In Senegal, a study investigating the relationship between SEP and large-scale door-to-door SMC delivery found no significant variation in SMC coverage across socioeconomic quintiles but revealed inequitable use of bed nets, with lower proportions of net use reported in the poorest households [24]. These studies underscore the critical influence of SEP on malaria prevention outcomes and highlight the heterogeneity in SEP measurement approaches.

Unlike other low-income countries that benefit from established demographic household surveys or poverty assessment tools with directly usable SEP indicators [25, 26], South Sudan faces significant data gaps for program monitoring and evaluation. National-level surveys that include wealth indices are scarce and outdated; for example, *the 2008 Population and Housing Census* (conducted prior to independence) remains the most recent large-scale demographic dataset [27–29]. The data gaps present a significant challenge to understanding the role of SEP in implementation and effectiveness of malaria control programs amidst acute multidimensional poverty, such as in South Sudan. Therefore, we developed a health intervention-specific set of asset ownership questions tailored to the South Sudanese context. These questions were piloted within routine cross-sectional surveys conducted as part of the monitoring and evaluation of the 2022 SMC implementation in targeted areas of South Sudan. Drawing on data from the 2022 SMC surveys, this study aimed to characterize household wealth through the construction of a contextually relevant asset-based index, and subsequently to investigate the association between household wealth and critical malaria prevention indicators, including SMC implementation, children malaria infection, and household malaria prevention practices.

## Methods

### Study design and setting

**Fig. 1:**
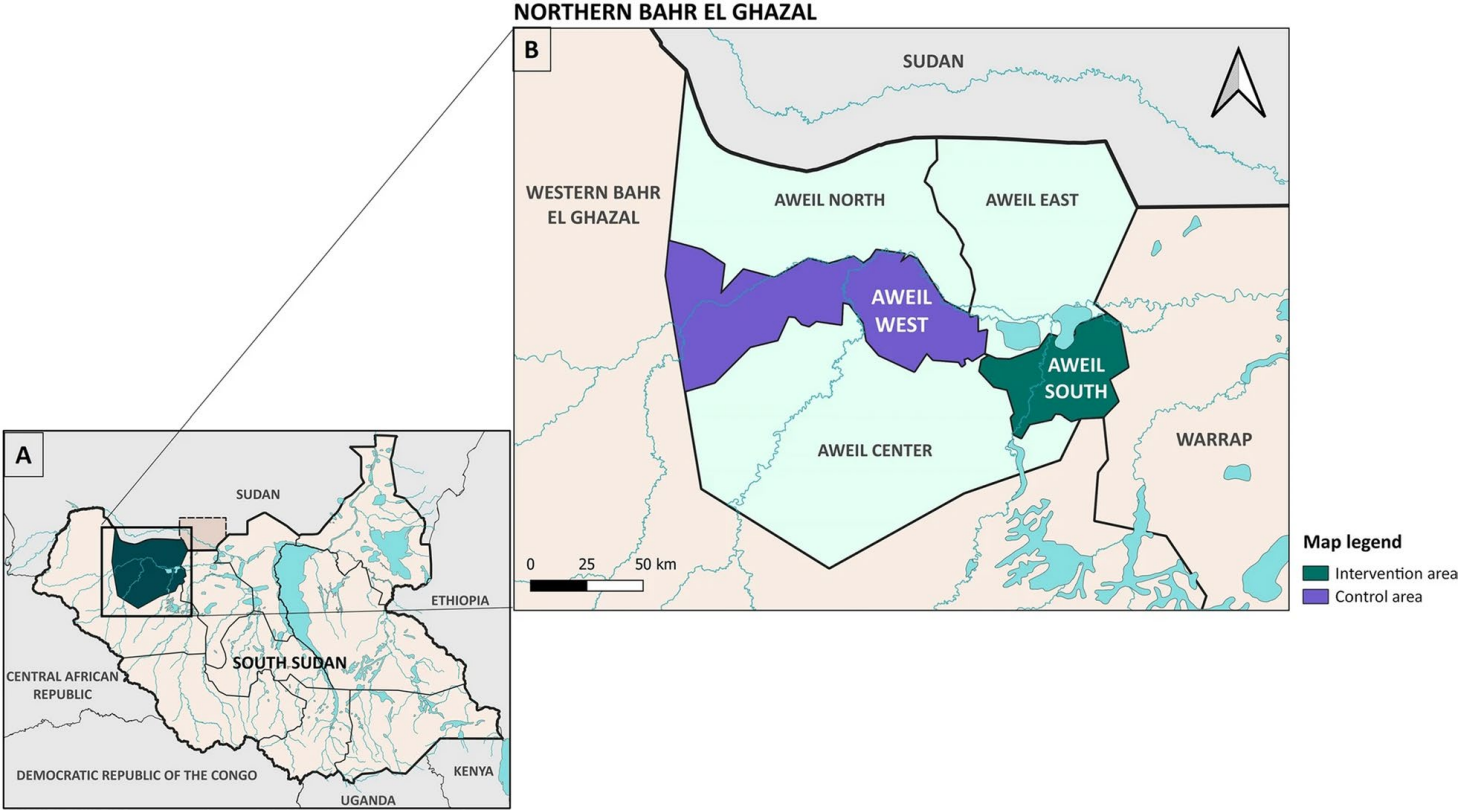
Map of Northern Bahr el Ghazal region in South Sudan (**A**) and the study counties (**B**). From [9]

This cross-sectional study was conducted in Northern Bahr el Ghazal state, South Sudan, utilizing repeated cross-sectional surveys of households with children aged 3–59 months during the 2022 SMC implementation. Northern Bahr el Ghazal, situated in northwest South Sudan, comprises five counties. For the 2022 SMC implementation, Aweil South County served as the intervention area and Aweil West County as the control area (Fig. 1). This study was embedded within a multi-component implementation research project investigating the effectiveness, feasibility, and acceptability of SMC in South Sudan [9].

**Fig. 2:**
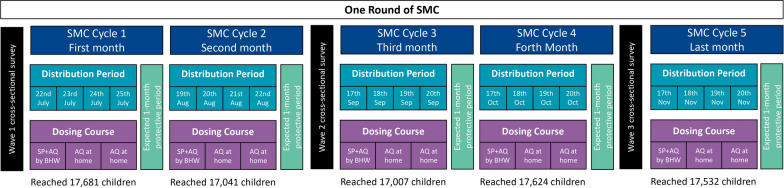
Timeline of SPAQ Administration during the SMC implementation in 2022 in South Sudan. *SMC* seasonal malaria chemoprevention, *SP* sulfoxide-pyrimethamine, *AQ* amodiaquine, *BHW* boma health worker

SMC and related community sensitization activities, supervised by the National Malaria Control Program of South Sudan and implemented with support from Malaria Consortium, were delivered through five consecutive monthly cycles, from July to November, aligning with the five continuous rainy months of the high malaria transmission season [9]. In each cycle, *boma* health workers (BHWs) conducted door-to-door household visits to administer Day 1 dose of SPAQ to eligible children, observe its full ingestion, instruct primary caregivers on administering the Day 2 and Day 3 AQ doses at home without BHW supervision to complete the 3-day administration course, and engage with household members to strengthen understanding of SMC (Fig. 2). Eligibility criteria for SMC administration in South Sudan required that children be aged 3–59 months, reside in the intervention area, and have no fever or malaria-related symptoms within the 48 h before administration. Children were considered ineligible if they had known allergies or adverse reactions to SP or AQ, had received anti-malarial medication or treatment with azithromycin or AQ in the prior 28 days, had severe malnutrition, or were Human immunodeficiency virus (HIV)-positive.

### Data collection and participant sampling

Three cross-sectional survey waves were designed to assess the effectiveness of SMC in preventing clinically significant cases of malaria among children aged 3–59 months. Wave 1 occurred in July 2022, covering the period before the first SMC delivery cycle in Aweil South and the onset of the rainy season (7 June to 7 July). Wave 2 and Wave 3 cross-sectional surveys were conducted in September and November 2022, respectively, covering the periods immediately following the start of SMC delivery in cycles 2 (18 August to 17 September) and 4 (17 October to 16 November) (Fig. 2).

Surveys were administered by pairs of data collectors using SurveyCTO (version 2.80) on mobile devices [30]. In each household, a roster of all children aged 3–119 months was compiled; from this list, SurveyCTO randomly selected one eligible child aged 3–59 months. The survey encompassed a variety of questions related to the selected child, the primary caregiver, the head of household, and the housing quality. Written informed consent was obtained from caregivers and heads of household prior to survey participation.

A multi-stage random sampling approach was used to select twenty households from each *boma*, based on lists provided by local registrars that recorded all current residents by household in both the intervention area (Aweil South) and the control area (Aweil West). The target sample size was set at 920 households for each survey across all 23 *bomas* in the intervention county (460 households) and 23 of the 31 *bomas* in the control county (460 households), selected independently for each survey wave using a simple random method due to lack of data on community population sizes before the surveys. This sample size was calculated based on a non-randomized controlled study to achieve 80% statistical power to detect, at a 5% significance level using a two-tailed test, a 40% or greater difference in malaria incidence between the intervention and control areas (assumed to be 0.05 cases per month per child) [9]. A total of 2767 households with children aged 3–59 months over the three survey waves (946 in Wave 1, 907 in Wave 2, and 914 in Wave 3) in both study counties had complete data and were included in the analysis. Post-hoc survey weights were generated based on community population sizes extracted from a county sampling frame, made available after the surveys, to ensure representativeness of analysis results. 

### Variables

#### Outcome measures

Surveys acquired information on SMC implementation, malaria infection in children, and household malaria prevention practices from household surveys across three waves. Outcomes related to SMC implementation included household visits by BHWs in the previous cycle, receipt of Day 1 SPAQ in the previous cycle, and caregiver knowledge of SMC age eligibility (“under five years” was considered a correct response) and caregiver awareness of the purpose of SMC (“to protect children against malaria during the high-risk season” or similar responses were considered correct) based on spontaneous responses. Outcomes related to children’s malaria infection included caregiver-reported fever in the previous one-month period and caregiver-reported RDT-confirmed malaria in the previous one-month period. Household malaria prevention practices were assessed using indicators including household ownership of mosquito nets, children sleeping under nets the night before the survey, and utilization of other malaria prevention tools the night before the survey (e.g., topical insecticide, insect repellant, and mosquito coils). All outcomes of interest were operationalized as binary variables.

#### Household wealth measure

A 12-item wealth score scale indicating household SEP from a wide range of asset items was generated through an iterative multi-step process to assign each household a weighted score according to the number of assets present. Households' SEP were then categorized into five quintiles.

#### Demographic variables

Demographic factors measured for each household across three study waves included children’s age, children’s sex, caregiver’s age, caregiver’s sex, caregiver’s partnership status, caregiver’s literacy, caregiver’s educational attainment, and caregiver’s occupation. Caregiver’s partnership status was classified as either partnered or unpartnered (including single, divorced, or widowed). Caregiver’s self-reported literacy was assessed based on the ability to read and write with understanding a short, simple statement about everyday life at the time of the household interview, and was operationalized as a binary variable. Detailed categories for demographic variables are presented in Table 2.

### Statistical analysis

#### Development of household wealth index

In Wave 1, a household wealth index was developed comprising 25 items related to assets, utilities, and infrastructure, operationalized as binary variables. The full description of survey questions for the asset items is available in supplementary Table S1. The selection process began by extracting initial variables from the 2008 South Sudan Population and Housing Census, retaining only those wealth items with a household ownership prevalence of 1% or greater [28, 29]. Additional variables were incorporated from Ballon and Duclos’ assessment of multidimensional poverty in South Sudan [27]. Moreover, variables from Simple Poverty Scorecards for Chad [31], Burkina Faso [32], Togo [33], and Nigeria [34] were then integrated, excluding items related to language, household size, overcrowding, or occupation, but including housing quality variables coded as binary categories [25]. Recognizing the local context of Aweil as an inland agricultural area, items specific to livestock ownership—including cattle, sheep, and goats—were included as binary categories. This systematic approach resulted in a comprehensive list of 25 items for the Wave 1 survey (Table S1).

Our initial analysis of the Wave 1 survey data indicated very low ownership of certain items and a disproportionately high ownership rate of others (Table S2). Additionally, the barter economy is still practiced in study localities alongside the introduction of a monetary market economy in South Sudan. Therefore, we refined our item selection by reducing the number of items using Mokken scaling and weighted items by multiple correspondence analysis (MCA) for subsequent Waves 2 and 3 surveys. While the initial Mokken scaling on the 25 items from Wave 1 informed our methodology and the refinement of the asset item list, the final 12 item wealth index scale used for outcome analysis in Waves 2 and 3 was constructed based on the refined set of 19 items collected during those later waves. The approach, combining Mokken scaling for item selection and MCA for weighting, was chosen for its suitability in constructing a wealth index from binary asset ownership data particularly in low-income settings where data distribution may not fully meet the assumptions of parametric methods like principal components analysis, which is typically used for index construction. This non-parametric approach has been validated as a measure of household SEP in low-income settings such as Vietnam [35]. Specifically, Mokken scale analysis is a data dimension reduction technique employing non-parametric item response theory approach using a monotone homogeneity model [36, 37]. The monotone homogeneity model was selected over the double monotonicity model due to violations of the assumption of non-intersection of the item step response. Mokken scale analysis has less restrictive assumptions than parametric item response theory; the assumptions of this model are unidimensionality (all items are related to one latent variable), monotonicity (probability of endorsing a response category based on presence of the latent trait remains the same or increases as the value of the latent trait increases), and local independence (answers on items depend solely on the latent trait and not on other characteristics of the individual or their environment) [38, 39]. 

#### Association of household SEP with SMC implementation, children malaria infection, and malaria prevention practices outcomes

Post-sampling weights were applied throughout this study. We described the socioeconomic and demographic characteristics of study participants (child, primary caregiver, and household) in the intervention (Aweil South) and control area (Aweil West) by wave using unweighted frequency and weighted percentages. Weighted chi-squared tests were performed to assess variable balance between counties across the three waves.

The household wealth scores derived from the 12-item wealth index scale were then operationalized as quintiles. We assessed the association between household wealth quintile and the following outcomes: child malaria infection, household malaria prevention practices, and SMC implementation during SMC implementation (Waves 2 and 3) using weighted chi-squared tests. Analysis of outcomes directly related to SMC implementation (household visits by BHWs, receipt of Day 1 SPAQ, and caregiver knowledge of SMC) was restricted to the intervention county (Aweil South). Weighted logistic regression adjusted for the caregiver’s gender, age, and education was performed to assess associations between wealth quintiles and outcomes, with results expressed as odds ratio (*OR*) and 95% confidence interval (95% *CI*). All statistical tests were two-tailed, and *P* < 0.05 was considered statistically significant.

Data were analyzed using Stata 17.0 (StataCorp, College Station, TX, USA) and figures were generated using R 3.4.2 (R Foundation for Statistical Computing, Vienna, Austria). Mokken scaling analysis was conducted with the “msp” package in Stata [38].

## Results

### Distribution of household ownership of asset-items

The descriptive analyses of household item ownership between the intervention and control area in Wave 3 are summarized in Table 1. The corresponding descriptive analyses for Wave 1 and Wave 2 are available in supplementary Tables S2 and S3.

**Table 1:** Household asset ownership in intervention (Aweil South) and control (Aweil West) areas during Wave 3

Item	Overall		Aweil South		Aweil West		*χ*^2^*	*P*
	*N* = 914		*N* = 457		*N* = 457			
	Yes	No	Yes	No	Yes	No		
Electricity								
* n*	19	895	7	450	12	445	2.951	0.094
%	2.2%	97.8%	1.2%	98.8%	2.9%	97.1%		
Television								
* n*	7	907	2	455	5	452	2.400	0.062
%	0.8%	99.2%	0.3%	99.7%	1.2%	98.8%		
Refrigerator								
* n*	7	907	2	455	5	452	1.214	0.349
%	1.0%	99.0%	0.6%	99.4%	1.3%	98.7%		
Wristwatch								
* n*	102	812	36	421	66	391	1.880	0.192
%	9.0%	91.0%	7.5%	92.5%	10.1%	89.9%		
Bed								
* n*	541	373	202	255	339	118	52.561	< 0.001
%	53.5%	46.5%	38.0%	62.0%	65.6%	34.4%		
Bank account								
* n*	18	896	9	448	9	448	1.807	0.262
%	2.5%	97.5%	3.2%	96.8%	1.9%	98.2%		
Cupboard/chest of drawers								
* n*	23	891	14	443	9	448	0.791	0.425
%	2.6%	97.4%	3.2%	96.8%	2.2%	97.8%		
Improved construction materials								
* n*	53	861	38	419	15	442	5.153	0.042
%	5.2%	94.8%	7.1%	92.9%	3.7%	96.3%		
Toilet facility								
* n*	347	567	173	284	174	283	1.871	0.172
%	38.6%	61.4%	35.8%	64.2%	40.7%	59.3%		
Improved roofing materials								
* n*	59	855	43	414	16	441	5.165	0.039
%	5.7%	94.4%	7.6%	92.4%	4.1%	95.9%		
Improved flooring materials								
* n*	44	870	34	423	10	447	3.778	0.052
%	4.1%	95.9%	5.7%	94.3%	2.8%	97.2%		
Fitted door								
* n*	270	644	116	341	154	303	0.222	0.681
%	30.7%	69.3%	29.9%	70.2%	31.3%	68.7%		
**Bucket**								
* n*	441	473	217	240	224	233	1.301	0.309
%	50.8%	49.2%	53.0%	47.1%	49.1%	50.9%		
**Soap**								
* n*	638	276	299	158	339	118	0.100	0.786
%	67.7%	32.3%	67.1%	32.9%	68.1%	31.9%		
**Pen or pencil**								
* n*	477	437	200	257	277	180	17.423	< 0.001
%	50.5%	49.6%	42.6%	57.4%	56.5%	43.5%		
**Blanket**								
* n*	406	508	188	269	218	239	0.526	0.468
%	43.8%	56.2%	42.3%	57.7%	45.0%	55.1%		
**Metal cooking pot**								
* n*	594	320	227	230	367	90	35.741	< 0.001
%	63.1%	36.9%	49.9%	50.1%	73.3%	26.7%		
**Metal tool**								
* n*	151	763	69	388	82	375	2.016	0.156
%	15.9%	84.1%	13.8%	86.2%	17.5%	82.5%		
Motorized vehicle								
* n*	94	820	33	424	61	396	2.984	0.127
%	10.6%	89.4%	8.6%	91.4%	12.1%	87.9%		

*n* number of respondents, % weighted percentage. Simpler items newly introduced into Wave 2 survey were bolded. Metal tools excluded knives or cooking utensils

^*^χ^2^ was calculated using weighted chi-squared tests

Mokken scale analysis was conducted on the 25 items in the Wave 1 survey, using a Loevinger's coefficient (H_i_) cut-off value of 0.3, which is conventionally used to reject unscalable items. This automated item selection procedure identified two preliminary scales: a seven-item scale related to personal use (vehicle, wristwatch, bank account, refrigerator, cupboard, cabinet or chest of drawers, electricity, television) and a five-item scale related to housing quality (bed, improved wall, improved toilet, improved roof, improved floor). For these 12 items selected in Wave 1, the single-item H_i_ values ranged from 0.34 to 0.79, with six items exceeding a value of 0.4, indicative of potentially "medium" to “strong” items [35].

We introduced seven simpler, context-specific items (bucket, soap, pen or pencil, blanket, metal cooking pot, metal tool) that could be easily verified by interviewers and answered by interviewees during the Wave 2 survey [27]. This adjustment addressed the limitations identified in the Wave 1 analysis, where many original items exhibited either very low or disproportionately high ownership prevalence, reducing their ability to differentiate between households effectively (Table S2). By including these additional items, we aimed to improve the sensitivity and applicability of the scale for capturing socioeconomic variability in the study context. Using Mokken scale analysis, we identified 12 items as the most scalable for inclusion in the scale out of a total of 19. The 12 items were motorized vehicle, wristwatch, bank account, cupboard, cabinet or chest of drawers, bucket, soap, pen or pencil, metal cooking pot, metal tool (scale 1), and electricity, television, and refrigerator (scale 2). The single-item Hi values for this final 12-item scale ranged from 0.36 to 0.66, with eleven items having Hi values exceeding 0.4, indicating potentially "medium" to “strong” items [35].

Cronbach’s alpha was used as a measure of the reliability of the 12-item wealth score scale. The items were weighted using the loadings from MCA from the first dimension, which explained 90% of the variance in the presence of scale items, prior to calculating Cronbach’s alpha [40]. The use of weights ensured that each wealth scale was evaluated based on its adjusted item scores. The wealth score scale based on these 12 items yielded a Cronbach’s alpha of 0.72, indicating ‘good’ internal validity. Based on this established reliability, the same 12-item scale was consistently applied to calculate the wealth index quintiles for each household surveyed in both Wave 2 and Wave 3 outcome analyses, ensuring comparability across waves.

**Fig. 3:**
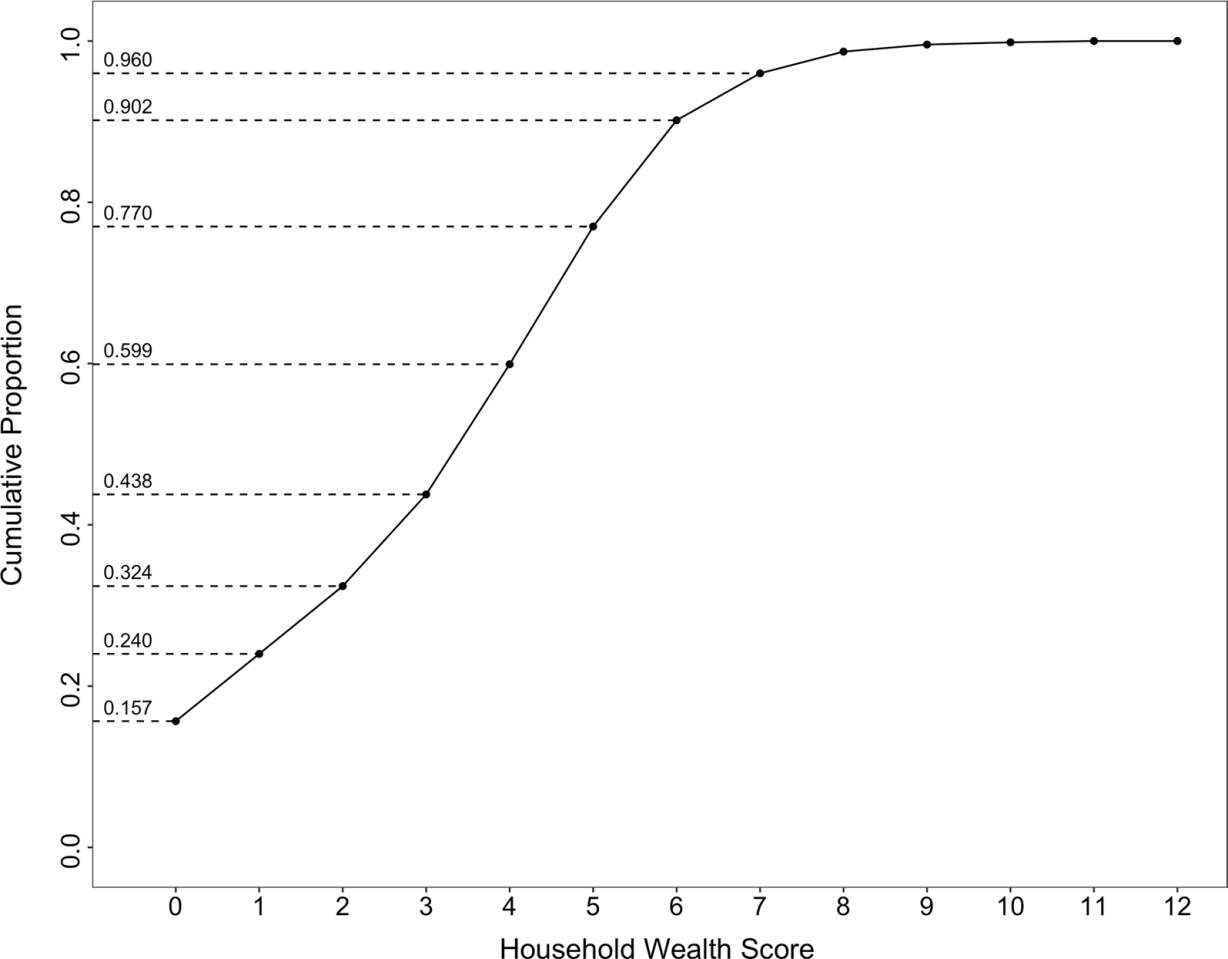
Cumulative proportion plot of the distribution of wealth score based on household ownership of assets (Wave 2 and Wave 3 surveys)

The cumulative proportion of the unweighted wealth score by households based on the 12-item wealth score scale in the Wave 2 is shown in Fig. 3. For instance, the wealthiest 10% of households achieved scores of 6 or more. The broad and approximately even distribution of scores, without significant concentration at any single score, indicates the effective capacity of the 12-item wealth score scale to differentiate household SEP.

### Socioeconomic and demographic characteristics of study participants by survey wave

The socioeconomic and demographic characteristics of study participants in the intervention area (Aweil South) and the control area (Aweil West) in Wave 2 and Wave 3 of SMC implementation are presented in Tables 2 and 3.

**Table 2:** Characteristics of SMC-eligible children, caregivers and households in intervention (Aweil South) and control (Aweil West) areas during Wave 2

Variable	Category	Aweil South (*N* = 436)		Aweil West (*N* = 471)		*χ*^2^*	df	*P*	Total (*N* = 907)	
		*n*	%	*n*	%				*n*	%
Child										
Fever	Yes	239	53.18	442	93.60	66.5064	(1, 25)	< 0.001	681	75.69
	No	197	46.82	29	6.40				226	24.31
RDT-confirmed malaria	Yes	178	40.11	355	75.26	12.1999	(1.41, 35.21)	< 0.001	533	59.69
	No	15	3.65	10	22.97				25	2.6
	Unknown (no fever/did not test if fever)	243	56.24	106	1.77				349	37.71
Sex	Male	218	53.63	240	51.54	0.29	(1, 25)	0.595	458	52.47
	Female	218	46.37	231	48.46				449	47.53
Age	3–12 months	33	8.82	50	10.08	0.6805	(2.81, 70.16)	0.558	83	9.52
	1 year	61	13.95	74	16.1				135	15.15
	2 years	103	24.25	89	18.43				192	21.01
	3 years	114	24.39	107	21.39				221	22.72
	4 years	88	20.44	108	21.76				196	21.18
	5 years	37	8.13	43	12.24				80	10.42
Caregiver										
Age, years	Under 20	60	10.47	65	21.46	1.2186	(1.99, 49.70)	0.304	125	16.59
	20–29	160	38.96	201	39.17				361	39.07
	30–39	167	38.53	147	27.27				314	32.26
	40–49	41	9.99	46	9.82				87	9.89
	50 or more above	8	2.06	12	2.28				20	2.18
Sex	Male	109	22.93	106	22.99	0.0001	(1, 25)	0.9925	215	22.96
	Female	327	77.07	365	77.01				692	77.04
Partnership status	Married/partnered	410	94.19	413	82.4	6.4301	(1, 25)	0.0178	823	87.63
	Non-partnered	26	5.81	58	17.6				84	12.37
Literacy	Yes	212	40.12	242	55.02	1.2915	(1, 25)	0.2665	454	48.42
	No	224	59.88	229	44.98				453	51.58
Education	None	294	65.28	198	42.52	5.9287	(1.92, 48.00)	0.006	492	52.61
	Informal/religious	83	21.9	73	12.23				156	16.52
	Primary or above	59	12.82	200	45.24				259	30.88
Occupation	Non-employed	73	12.32	61	13.22	1.0461	(1.76, 43.91)	0.352	134	12.82
	Unemployed	35	8.49	33	7.9				68	8.16
	Agricultural	316	76.51	311	67.39				627	71.43
	Unskilled manual work	2	0.77	33	6.92				35	4.19
	Skilled/Service/professional	10	1.91	33	4.57				43	3.39
Caregiver knowledge of SMC purpose	Yes	390	91.88						390	91.88
	No	35	8.12						35	8.12
Caregiver knowledge of SMC eligibility	Yes	399	93.89						399	93.89
	No	26	6.11						26	6.11
Household										
Net ownership	Yes	289	64.67	346	73.98	0.4883	(1, 25)	0.491	635	69.86
	No	147	35.33	125	26.02				272	30.14
Selected child used mosquito net night before survey	Yes	270	58.68	336	69.58	0.7406	(1, 25)	0.398	606	64.75
	No	166	41.32	135	30.42				301	35.25
Household use of other malaria prevention	Yes	95	18.36	41	8.38	4.1793	(1, 25)	0.052	136	9.59
	No	341	81.64	430	91.62				771	90.41
Wealth index	1st quintile (poorest)	97	18.26	73	19.87	2.1093	(2.46, 61.53)	0.120	170	19.16
	2nd quintile	141	30.5	81	16.76				222	22.85
	3rd quintile	100	27.65	103	18.1				203	22.33
	4th quintile	61	14.56	145	32.01				206	24.27
	5th quintile (wealthiest)	37	9.03	69	13.27				106	11.39
Household visit by SMC BHWs	Yes	399	92.34						399	92.34
	No	37	7.66						37	7.66
Receipt of Day 1 SPAQ	Yes	399	91.96	113	23.62	59.3232	(1, 25)	< 0.001	512	53.9
	No	37	8.04	358	76.38				395	46.1

*n* number of respondents, % weighted percentage,* BHW* *boma* health workers, *SPAQ* sulfadoxine-pyrimethamine and amodiaquine, *RDT* rapid diagnostic test, *SMC* seasonal malaria chemoprevention. Blank cells indicate no intervention in Aweil West (control region), therefore no corresponding survey variables

^*^χ^2^ was calculated using weighted chi-squared tests

**Table 3:** Characteristics of SMC-eligible children, caregivers and households in intervention (Aweil South) and control (Aweil West) areas during Wave 3

Variable	Category	Aweil South (*N* = 457)		Aweil West (*N* = 457)		*χ*^2^*	df	*P*	Total (*N* = 914)	
		*n*	%	*n*	%				*n*	%
Child										
Fever	Yes	250	53.4	405	88.29	10.4333	(1,24)	0.0036	655	73.03
	No	207	46.6	52	11.71				259	26.97
RDT-confirmed malaria	Yes	183	39.24	353	73.63	10.7761	(1.25, 29.90)	0.001	536	58.59
	No	11	2.79	5	1.06				16	1.82
	Unknown (no fever/did not test if fever)	263	57.97	99	25.31				362	39.59
Sex	Male	228	50.75	216	45.62	2.0575	(1,24)	0.1644	444	47.86
	Female	229	49.25	241	54.38				470	52.14
Age	3–12 months	12	4.1	34	8.78	0.7798	(3.07, 73.66)	0.512	46	6.73
	1 year	100	21.32	89	19.14				189	20.09
	2 years	118	23.37	108	23.73				226	23.57
	3 years	118	23.36	112	21.48				230	22.3
	4 years	87	22.14	83	18.22				170	19.93
	5 years	22	5.72	31	8.65				53	7.37
Caregiver										
Age, years	Under 20	78	15.42	82	21.24	0.8062	(2.46, 59.03)	0.474	160	18.7
	20–29	212	48.46	201	39.5				413	43.42
	30–39	125	27.26	120	26.67				245	26.93
	40–49	35	7.38	31	7.52				66	7.46
	50 or more above	7	1.48	23	5.06				30	3.5
Sex	Male	107	21.12	98	25.78	0.2719	(1,24)	0.6069	205	23.78
	Female	350	78.88	359	74.22				709	76.22
Partnership status	Married/partnered	417	91.18	388	80.87	2.3391	(1,24)	0.1392	805	85.38
	Non-partnered	40	8.82	69	19.13				109	14.62
Literacy	Yes	233	46.53	222	47.24	0.0059	(1,24)	0.9396	455	46.93
	No	224	53.47	235	52.76				459	53.07
Education	None	305	63.11	199	45.73	3.0125	(1.42, 34.13)	0.078	504	53.33
	Informal/religious	94	21.96	78	14.82				172	17.94
	Primary or above	58	14.93	180	39.45				238	28.73
Occupation	Non-employed	89	17.57	54	14.96	1.1631	(2.23, 53.47)	0.324	143	16.1
	Unemployed	43	7.86	33	8.39				76	8.16
	Agricultural	316	71.7	316	65.41				632	68.16
	Unskilled manual work	0	0	24	3.73				24	2.1
	Skilled/Service/professional	9	2.87	30	7.52				39	5.49
Caregiver knowledge of SMC purpose	Yes	413	95.99						413	95.99
	No	24	4.01						24	4.01
Caregiver knowledge of SMC eligibility	Yes	415	96.21						415	96.21
	No	22	3.79						22	3.79
Household										
Net ownership	Yes	349	76.22	338	71.22	0.1184	(1,24)	0.734	687	73.41
	No	108	23.78	119	28.78				227	26.59
Selected child used mosquito net night before survey	Yes	309	66.42	333	70.32	0.0643	(1,24)	0.802	642	68.62
	No	148	33.58	124	29.68				272	31.38
Household use of other malaria prevention night before survey	Yes	142	27.86	58	11.26	3.8094	(1,24)	0.063	200	18.52
	No	315	72.14	399	88.74				714	81.48
Wealth index	1st quintile (poorest)	117	23.23	59	20.61	0.5575	(1.79, 42.89)	0.558	176	21.76
	2nd quintile	106	22.95	74	13.75				180	17.77
	3rd quintile	92	24.62	120	22.55				212	23.45
	4th quintile	83	16.18	118	27.41				201	22.5
	5th quintile (wealthiest)	59	13.02	86	15.68				145	14.52
Household visit by SMC BHWs	Yes	431	95.07						431	95.07
	No	26	4.93						26	4.93
Receipt of Day 1 SPAQ	Yes	430	94.75	71	14.15	93.1634	(1,24)	< 0.001	501	49.4
	No	27	5.25	386	85.85				413	50.6

*n* number of respondents, % weighted percentage.* BHW* *boma* health workers, *SPAQ* sulfadoxine-pyrimethamine and amodiaquine, *RDT* rapid diagnostic test,* SMC* seasonal malaria chemoprevention. Blank cells indicate no intervention in Aweil West (control region), therefore no corresponding survey variables

^*^χ^2^ was calculated using weighted chi-squared tests

Descriptive analysis of the wealth index based on the 12-item wealth score scale from Wave 2 showed a higher weighted proportion of households in the 4th quintile (32.01%, 145/471) and 5th quintile (13.27%, 69/471) in Aweil West compared to Aweil South (4th quintile: 14.56%, 61/436; 5th quintile: 9.03%, 37/436), though this difference was not statistically significant (*P* = 0.120). Similar results were found in Wave 3 (Table 3).

### Association of household wealth index with SMC implementation, children malaria infection, and household malaria prevention practices

The chi-squared and regression results for associations between household wealth index, and SMC implementation, malaria infection in children, and household malaria prevention methods from Wave 2 and Wave 3 surveys are presented in Table 4.

**Table 4:** Logistic regression analysis of SMC implementation, children malaria infection, and household malaria prevention practices during Wave 2 and Wave 3 of seasonal malaria chemoprevention implementation

	Outcome	Wealth index	Analytic sample (*N*)	Yes (*n*)	%	No (*n*)	%	*χ*^2*^	*P*	*OR*	95% *CI*	*P*
Aweil South only												
SMC implementation	Household visits by SMC BHWs	1st quintile (poorest)	881	201	21.19	12	16.28	6.684	0.365	1.43	0.44–4.70	0.534
		2nd quintile		230	26.99	17	27.40			1.14	0.39–3.30	0.797
		3rd quintile		173	25.39	16	30.14			ref		
		4th quintile		127	14.76	14	23.08			0.80	0.47–1.35	0.378
		5th quintile (wealthiest)		94	11.66	2	3.09			4.01	0.35–45.77	0.245
	Selected child received Day 1 SPAQ in previous cycle	1st quintile (poorest)	881	205	21.39	8	12.58	7.463	0.345	2.01	0.57–7.16	0.259
		2nd quintile		229	26.76	18	31.31			1.01	0.30–3.44	0.973
		3rd quintile		173	25.43	16	29.76			ref		
		4th quintile		128	14.81	13	23.02			0.78	0.46–1.33	0.339
		5th quintile (wealthiest)		94	11.61	2	3.34			3.48	0.28–43.98	0.313
	Caregiver knowledge of the purpose of SMC	1st quintile (poorest)	862	192	20.28	16	26.13	7.461	0.448	0.77	0.10–5.77	0.783
		2nd quintile		215	26.02	18	31.69			0.85	0.29–2.46	0.754
		3rd quintile		171	26.36	17	30.33			ref		
		4th quintile		130	15.33	7	10.57			1.28	0.45–3.66	0.616
		5th quintile (wealthiest)		95	12.01	1	1.27			18.57	2.14–161.08	0.011
	Caregiver knowledge of the eligible age range for SMC	1st quintile (poorest)	862	189	19.70	19	38.53	13.025	0.155	0.33	0.04–2.32	0.246
		2nd quintile		219	26.14	14	30.68			0.64	0.24–1.69	0.346
		3rd quintile		178	26.89	10	21.04			ref		
		4th quintile		133	15.40	4	8.19			1.01	0.17–5.99	0.990
		5th quintile (wealthiest)		95	11.87	1	1.56			4.41	0.28–70.08	0.273
Aweil South and Aweil West Combined												
Malaria infection in children	Child experienced fever in previous month	1st quintile (poorest)	1821	222	19.30	124	23.83	17.521	0.432	0.66	0.26–1.70	0.378
		2nd quintile		273	18.70	129	24.95			0.66	0.36–1.20	0.164
		3rd quintile		328	23.62	87	20.79			ref		
		4th quintile		311	24.54	96	20.02			1.01	0.64–1.60	0.966
		5th quintile (wealthiest)		202	13.83	49	10.42			1.05	0.67–1.65	0.821
	Child experienced RDT-confirmed malaria in previous month	1st quintile (poorest)	1100	161	17.92	5	13.67	3.425	0.543	1.27	0.41–3.99	0.672
		2nd quintile		197	17.05	14	26.53			0.83	0.24–2.84	0.758
		3rd quintile		269	24.01	10	27.95			ref		
		4th quintile		262	25.45	7	19.85			1.42	0.70–2.93	0.319
		5th quintile (wealthiest)		180	15.58	5	11.99			1.13	0.49–2.63	0.765
Household malaria prevention practices	Household mosquito net ownership	1st quintile (poorest)	1821	135	9.81	211	47.37	408.891	< 0.001	0.12	0.05–0.26	< 0.001
		2nd quintile		262	17.81	140	26.60			0.34	0.21–0.55	< 0.001
		3rd quintile		345	26.69	70	13.32			ref		
		4th quintile		357	29.38	50	8.22			1.70	0.92–3.09	0.086
		5th quintile (wealthiest)		223	16.31	28	4.49			1.90	0.83–4.34	0.122
	Selected child used mosquito net night before survey	1st quintile (poorest)	1821	110	7.46	236	46.49	487.853	< 0.001	0.07	0.03–0.17	< 0.001
		2nd quintile		230	17.02	172	26.87			0.29	0.17–0.50	< 0.001
		3rd quintile		338	27.89	77	12.90			ref		
		4th quintile		350	30.63	57	8.87			1.60	0.92–2.77	0.092
		5th quintile (wealthiest)		220	17.00	31	4.88			1.61	0.82–3.17	0.159
	Household use of other malaria prevention night before survey	1st quintile (poorest)	1821	44	11.20	302	22.18	54.720	0.008	0.50	0.16–1.54	0.213
		2nd quintile		59	15.93	343	21.11			0.78	0.39–1.55	0.467
		3rd quintile		70	21.62	345	23.13			ref		
		4th quintile		80	26.60	327	22.79			1.43	0.88–2.32	0.138
		5th quintile (wealthiest)		83	24.64	168	10.79			2.75	1.30–5.83	0.010

*n* number of respondents,* %* weighted percentage. *BHW* *boma* health worker, *SPAQ* sulfadoxine-pyrimethamine and amodiaquine, *SMC* seasonal malaria chemoprevention, *RDT* rapid diagnostic test, *OR* odds ratio, *CI* confidence interval

^*^χ^2^ was calculated using weighted chi-squared tests. Full models were adjusted for caregiver’s age, sex, and education; adjusted odds ratios are reported

#### SMC implementation

In Aweil South County, no statistically significant association was found between wealth index and household visits by BHWs (*χ*^2^ = 6.684, *P* = 0.365) or receipt of Day 1 SPAQ (*χ*^2^ = 7.463, *P* = 0.345) in the one-month period preceding both Wave 2 and Wave 3 surveys. Similarly, no statistically significant association was found between wealth index and caregiver knowledge of SMC purpose (*χ*^2^ = 7.461, *P* = 0.448) or caregiver knowledge of SMC eligibility (*χ*^2^ = 13.025, *P* = 0.155). However, adjusted regression models suggested higher odds of primary caregivers in the 5th wealth quintile being aware of SMC's purpose compared with the 3rd quintile (*OR* = 18.57, 95% *CI:* 2.14–161.08, *P* = 0.011). Still, the regression results indicated that this set of SMC implementation outcomes was not significantly associated with household wealth index.

#### Children's malaria infection

We found no association between the household wealth index and caregiver-reported child fever or caregiver-reported RDT-confirmed malaria infection during the one-month period prior to both Wave 2 and Wave 3 surveys. For instance, children in the 1st (poorest) quintile had an *OR* of 0.66 (95% *CI:* 0.26–1.70, *P* = 0.378) for caregiver-reported fever compared to children in the 3rd (middle) wealth quintile. Similarly, children in the 5th (wealthiest) quintile showed an *OR* of 1.05 (95% *CI:* 0.67–1.65, *P* = 0.821). Likewise, for caregiver-reported RDT-confirmed malaria infection, the 1st quintile had an *OR* of 1.27 (95% *CI:* 0.41–3.99, *P* = 0.672) for RDT-confirmed malaria, while the 5th quintile had an *OR* of 1.13 (95% *CI:* 0.49–2.63, *P* = 0.765). However, comparing the results between all five quintiles, quintiles 1 and 2 have stronger association with fever (cumulative 0.542 *P*-value) compared with quintiles 4 and 5 (cumulative 1.787 *P*-value), suggesting an association between malaria and poverty.

#### Malaria prevention practices

The proportion of households owning mosquito nets varied significantly by wealth index quintile (*χ*^2^ = 408.891, *P* < 0.001). For example, among households without mosquito nets, only 4.49% were from the 5th quintile, while 47.37% were from the 1st quintile. The model results showed a statistically significant 90% reduction in the odds of net ownership in the 1st quintile relative to the 3rd quintile (*OR* = 0.12, 95% *CI:* 0.05–0.26, *P* < 0.001). A statistically significant effect size was observed when comparing the 2nd quintile with the 3rd quintile in terms of net ownership (*OR* = 0.34, 95% *CI:* 0.21–0.55, *P* < 0.001). We found higher odds of net ownership were found in the 4th quintile (*OR* = 1.70, 95% *CI:* 0.92–3.09, *P* = 0.086) and 5th quintile (*OR* = 1.90, 95% *CI:* 0.83–4.34, *P* = 0.122), although these associations were not statistically significant.

Results of chi-squared tests also showed significant variation in children sleeping under mosquito nets the night before the survey by wealth quintile (*χ*^2^ = 487.853, *P* < 0.001). Similar to the association between wealth index and mosquito net ownership, the results indicated a gradient effect. Lower wealth quintiles experienced a significant reduction in odds, while higher wealth quintiles showed a trend towards increased odds, although not statistically significant (*OR* = 0.07, 95% *CI:* 0.03–0.17, *P* < 0.001 in 1st quintile vs. 3rd quintile; *OR* = 1.60, 95% *CI:* 0.92–2.77, *P* = 0.092 in 4th quintile vs. 3rd quintile).

In terms of household use of other malaria prevention methods, the chi-squared test indicated a statistically significant difference across different wealth quintiles (*χ*^2^ = 54.720, *P* = 0.008). The model results showed that households in the 5th quintile had significantly higher odds of using other malaria prevention methods the night before the survey compared with those in the 3rd quintile (*OR* = 2.75, 95% *CI:* 1.30–5.83, *P* = 0.010).

## Discussion

Malaria is endemic across South Sudan, where control efforts are hampered by ongoing political, economic, and social challenges. In 2022, South Sudan implemented SMC for the first time under routine program conditions as part of the new *South Sudan’s National Malaria Strategic Plan 2020–2025* [8]. Although sparse studies have demonstrated that SEP is closely associated with malaria prevalence, incidence, case management, and prevention practices [21, 41–45], the association between SEP and SMC implementation remains largely understudied. This study is the first attempt to construct a wealth index as a proxy of household SEP for South Sudan to assess its impact on SMC and examine the association between SEP, children’s malaria infection, and household malaria prevention practices.

A 12-item wealth score scale was generated from a wide range of asset items using Mokken scale analysis; these 12 items allowed for distinct stratification of household SEP by wealth quintiles. We found no evidence that household wealth quintiles were associated with household access to SMC and children’s receipt of Day 1 SPAQ in Aweil South County (intervention area). This contrasts with findings from other maternal and child health studies that link lower SEP to reduced access to healthcare [46–48]. It could be argued that the door-to-door delivery method of SMC may have mitigated wealth-related disparities by providing equal opportunities for all SEP groups to access SMC passively and receive malaria prevention information. Supporting this, 93.7% of survey respondents (830/893) reported visits by BHWs during Wave 2 and Wave 3, and 99.15% of those received SMC medicine during these visits.

However, substantial disparities in malaria prevention practices by household wealth were observed. Households in lower wealth quintiles were significantly less likely to own mosquito nets and, consequently, less likely to have children sleeping under nets the night before the survey, a finding similar to a Senegal study conducted in the context of SMC [24]. A multi-country study in Angola, Tanzania, and Uganda also reported that while free bed net distribution can improve equity in bed net ownership and use, significant disparities persist across socioeconomic groups, with wealthier households generally benefiting more; and the association between net ownership also varied by country and changed over time [49]. South Sudan distributes standard long-lasting insecticidal nets (LLINs) through mass campaigns universally every three years (not affected by any factor other than insecurity and floods) [50]. However, poorer households sometimes sell LLINs in markets or use them for other purposes, such as recreational, decorative, fishing, or food or cattle protection. The observed “dose-response” effect between wealth-associated disparities and net use underscores that South Sudan has not yet achieved equity in mosquito net ownership across SEP groups. This study also reported a positive association between wealthier households and use of alternative malaria prevention tools, consistent with findings that the poorest SEP groups are the least able to purchase malaria prevention resources [51–54].

SMC has been shown to be an effective and feasible intervention in South Sudan [9]. Updated WHO guidelines on SMC have provided implementers greater latitude to adapt the intervention in terms of age eligibility, delivery methods, and geographic targeting [6]. It is crucial to ensure that the design of interventions is tailored to external factors that influence program implementation, outcomes, and impact, such as specific social, economic, infrastructural, and climatic challenges faced in South Sudan [46, 55]. These tailored approaches entail robust monitoring and evaluation mechanisms, which will be performed to track coverage, treatment adherence, and equitable access across socioeconomic and demographic groups and other hard-to-reach but vulnerable populations. This includes addressing implementation challenges through timely supervision, feedback loops, and adaptive strategies to improve program effectiveness in complex settings.

Currently, South Sudan mass LLIN campaigns are largely conducted by volunteers using paper-based systems to record distribution data via antenatal care and the Expanded Program on Immunization interventions [56]. Our findings demonstrate the need for efforts to reduce wealth-associated disparities in access to malaria prevention practices, such as mosquito nets. Acknowledging that observed inconsistencies in net use coverage and the repurposing of nets for other uses are often driven by underlying socioeconomic factors, such as the lack of basic necessities—issues typically beyond their direct mandate—addressing these challenges within the scope of net distribution campaigns requires improving net supply chains, expanding distribution outside immunization programs, and enhancing community knowledge of correct net usage [56]. Efforts to improve these systems are underway; for example, a digital tool introduced in 2022 in Northern Bahr el Ghazal has shown promise in improving data accuracy and enabling real-time monitoring during LLIN campaigns [57]. Additionally, recent LLIN campaigns have adopted flexible delivery models in South Sudan, such as combining door-to-door and fixed-point approaches, to address logistical challenges and better adapt to local circumstances, such as in flood-prone areas [58]. Notably, Pyrethroid-piperonyl butoxide (PBO) nets with advanced insecticidal properties are now preferred to standard LLINs due to their greater effectiveness against insecticide-resistant mosquitoes [4]; however, their higher costs pose challenges for widespread adoption, particularly among poorer households. On the other hand, wealthier households are more likely to purchase alternative mosquito nets that are often more decorative and less efficacious than PBO nets, while still being more expensive. This highlights the urgent need for direct investment in government-led, large-scale distribution of PBO nets, and to ensure equitable access [21, 59]. Integration of malaria vaccines with existing tools such as SMC or net distribution campaigns have the potential to further reduce gaps in malaria prevention coverage [60].

The key strength of this study is the development of a locally-tailored wealth index to measure household SEP in South Sudan, where standardized assessment tools are lacking and income data are unreliable due to irregular reporting and expenditure patterns. This index provides a practical and adaptable tool for socioeconomic assessment in settings with limited demographic and economic data. This study also includes updated wealth items to improve differentiation among households in lower wealth quintiles, addressing the challenges of wealth measurement in a predominantly barter-based economy. While Mokken scaling facilitated the reduction and refinement of asset-based items, the broader contribution lies in creating a contextually relevant wealth index for a fragile and underdeveloped setting, which can be adapted for use in similar regions.

General limitations of the study include its reliance on caregiver self-reporting for certain variables, including hard-to-observe asset items (e.g., bank account ownership) and malaria prevention practices, such as sleeping under a mosquito net. This reliance may introduce biases, such as social desirability bias and recall biases, which could affect the accuracy of responses. Specifically, social desirability bias may have led caregivers to potentially over-report engagement in recommended malaria prevention practices or ownership of assets perceived as indicators of higher socioeconomic status. Recall bias could potentially affect the accurate reporting of events over the past month, such as fever episodes or prevention tool use. While these biases are inherent to self-report data collection, several steps were taken to mitigate their potential impact. The presence of most asset items was independently verified by data collectors during household interviews, and potential recall bias was minimized by ensuring a short time gap between SMC delivery cycles and the surveys, adding confidence to the reliability of responses. Another limitation is the inability to compare our newly developed wealth index scale with other commonly used measures of household SEP, such as reliable household income or expenditure data. Such comparisons would provide valuable external validation and a more comprehensive understanding of the index’s applicability. However, collecting stable income and regular consumption data is particularly challenging in settings like South Sudan due to informal and irregular economic patterns, reflected in very limited access to formal banking systems (Table 1 shows only approximately 2.5% of households in our sample reported having a bank account). While our scale represents one dimension of SEP, its validity would be further strengthened through comparisons with other standardized measures in future studies where data collection is feasible [35]. Additionally, this study cannot make definitive statements regarding causative pathways through which household wealth influences SMC implementation outcomes or malaria prevention practices, due to the potential for mediation effects and unaccounted confounding factors [45, 61]. Finally, while the surveys in this study primarily focused on Northern Bahr el Ghazal, they provide valuable insights more generally into household SEP and its association with SMC implementation outcomes. The developed 12-item wealth score scale was tailored to capture socioeconomic variability within this region based on locally relevant assets. Given the vast geographic and economic diversity across South Sudan, including varying levels of urbanization, economic activities, and asset availability, generalizing an index developed in one region to other parts of the country is challenging and requires validation; its direct application to other states or counties would require careful consideration and ideally, re-validation using local data.

## Conclusions

This study successfully developed and applied a locally tailored wealth index using Mokken scaling to characterize household wealth in South Sudan. While household wealth inequality was evident in the use of self-purchasing or active malaria prevention tools, this disparity was notably absent in access to door-to-door SMC delivery. This demonstrates the potential of targeted delivery methods to mitigate wealth-related barriers to essential health interventions in fragile contexts. Ultimately, fostering a more equitable malaria control environment in settings like South Sudan necessitates continued efforts to improve overall socioeconomic conditions and ensure accessible mass distribution of integrated malaria prevention tools.

## Supplementary Information

Additional file1 (DOCX 53 KB)

## Data Availability

The data that support the findings of this study are available on reasonable request from the corresponding author.

## Abbreviations

AQ: Amodiaquine
BHW: *boma* health worker
*CI*: Confidence interval
HIV: Human immunodeficiency virus
LLIN: Long-lasting insecticidal net
MCA: multiple correspondence analysis
*OR*: Odds ratio
PBO: Pyrethroid-piperonyl butoxide
RDT: Rapid diagnostic test
SEP: Socioeconomic position
SMC: Seasonal malaria chemoprevention
SP: Sulfadoxine-pyrimethamine
USD: United States dollars
WHO: World Health Organization
