# Supplementary material for: Development of a new wealth index for South Sudan: association between household wealth and malaria prevention practices in the context of seasonal malaria chemoprevention in Northern Bahr el Ghazal, South Sudan
Source: Infect Dis Poverty. 2025 Jul 1;14:57. doi: 10.1186/s40249-025-01327-3 (PMC12210560; doi:10.1186/s40249-025-01327-3)
Supplement: Supplementary file 1 — Additional file1 (DOCX 53 KB) [file 40249_2025_1327_MOESM1_ESM.docx]

# Supplementary materials

Table S1. Operational survey questions of household ownership of asset-items in Wave 1 of seasonal malaria chemoprevention

| Field | Question | Response |
| --- | --- | --- |
| Electricity | Does the household have access to electricity? | Yes / No |
| Radio | Does the household have a working radio? | Yes / No |
| Television | Does the household have a working television? | Yes / No |
| Mobile telephone | Does the household have a working telephone or mobile telephone? | Yes / No |
| Refrigerator | Does the household have a working refrigerator? | Yes / No |
| Agricultural land | Does the household own agricultural land? | Yes / No |
| Wristwatch | Do any of the household members own a wristwatch? | Yes / No |
| Cattle | Does the household own cattle (i.e. cows/oxen)? | Yes / No |
| Bed | Does the household own one or more beds? | Yes / No |
| Bicycle | Does the household own a usable bicycle? | Yes / No |
| Goats or sheep | Does the household own goats or sheep? | Yes / No |
| Bank account | Do any of the household members have a bank account? | Yes / No |
| Table | Does the household own a table? | Yes / No |
| Cupboard/chest of drawers | Does the household own a cupboard, cabinet or chest of drawers? | Yes / No |
| Water source | Does the household obtain water from an ‘improved’ source?  ‘Improved’ includes sources such as bottled water, piped water, or protected wells. | Yes / No |
| Cooking source | Does the household use an ‘improved’ fuel source for cooking?  ‘Improved’ includes electricity, LPG, biogas, kerosene, coal, or charcoal. | Yes / No |
| Shoes/sandals | Does each household member have at least one pair of shoes or sandals? | Yes / No |
| Overcrowding | Is the household overcrowded?  Overcrowding is defined as more than three people per habitable room. | Yes / No |
| Lighting | Does the household use ‘improved’ lighting?  ‘Improved’ includes electricity, LPG/gas, or solar power. | Yes / No |
| Improved construction materials | Are the walls of the house built from an ‘improved’ material?  ‘Improved’ includes stone, brick, concrete, cement blocks, or wood planks. | Yes / No |
| Toilet facility | Does the household have toilet to sewer, ventilated pit latrine, or simple pit latrine? | Yes / No |
| Improved roofing materials | Is the roof of the house built from an ‘improved’ material?  ‘Improved’ includes metal, ceramic tiles, cement, or roofing shingles. | Yes / No |
| Improved flooring materials | Is the floor of the house built from an ‘improved’ material?  ‘Improved’ includes tiles, cement, polished wood, or vinyl tiles. | Yes / No |
| Motorized vehicle | Does the household own or have exclusive access to a car, truck, motorbike, autorickshaw/Raksha/Bajaj or motorized scooter?  This question combines two separate questions: one regarding car/truck ownership and the other ownership of a motorbike, autorickshaw/Raksha/Bajaj or motorized scooter. | Yes / No |

Table S2. Household asset ownership in intervention (Aweil South) and control (Aweil West) areas during Wave 1

|  |  | Overall | | Aweil South | | Aweil West | | *χ*²* | *P* |
| --- | --- | --- | --- | --- | --- | --- | --- | --- | --- |
|  |  | N = 946 | | N = 463 | | N = 483 | |  |  |
| Item |  | Yes | No | Yes | No | Yes | No |  |  |
| Electricity | *n* | 8 | 938 | 3 | 460 | 5 | 478 | 3.533 | 0.194 |
|  | % | 1.4% | 98.6% | 0.7% | 99.3% | 2.1% | 97.9% |  |  |
| Radio | *n* | 69 | 877 | 7 | 456 | 62 | 421 | 51.105 | < 0.001 |
|  | % | 7.4% | 92.5% | 1.3% | 98.7% | 13.5% | 86.5% |  |  |
| Television | *n* | 4 | 942 | 1 | 462 | 3 | 480 | 0.392 | 0.669 |
|  | % | 0.7% | 99.3% | 0.5% | 99.5% | 0.9% | 99.1% |  |  |
| Mobile telephone | *n* | 368 | 578 | 121 | 342 | 247 | 236 | 121.381 | < 0.001 |
|  | % | 40.7% | 59.3% | 23.0% | 77.0% | 58.0% | 42.0% |  |  |
| Refrigerator | *n* | 1 | 945 | 0 | 463 | 1 | 484 | 1.389 | 0.325 |
|  | % | 0.2% | 99.9% | 0.0% | 100.0% | 0.3% | 99.7% |  |  |
| Agricultural land | *n* | 819 | 127 | 426 | 37 | 393 | 90 | 25.138 | < 0.001 |
|  | % | 85.6% | 14.4% | 91.3% | 8.7% | 80.0% | 20.1% |  |  |
| Wristwatch | *n* | 78 | 868 | 30 | 433 | 48 | 435 | 5.764 | 0.051 |
|  | % | 9.2% | 90.8% | 6.9% | 93.1% | 11.4% | 88.6% |  |  |
| Cattle | *n* | 318 | 628 | 201 | 262 | 117 | 366 | 34.683 | < 0.001 |
|  | % | 32.0% | 68.0% | 41.0% | 59.0% | 23.1% | 76.9% |  |  |
| Bed | *n* | 632 | 314 | 225 | 238 | 407 | 76 | 171.623 | < 0.001 |
|  | % | 62.0% | 38.0% | 41.4% | 59.7% | 82.2% | 17.8% |  |  |
| Bicycle | *n* | 82 | 864 | 30 | 433 | 52 | 431 | 2.071 | 0.248 |
|  | % | 7.9% | 92.1% | 6.7% | 93.3% | 9.1% | 90.9% |  |  |
| Goats or sheep | *n* | 461 | 485 | 234 | 229 | 227 | 256 | 0.160 | 0.750 |
|  | % | 46.7% | 53.3% | 47.4% | 52.6% | 45.9% | 54.1% |  |  |
| Bank account | *n* | 9 | 939 | 3 | 460 | 6 | 479 | 1.341 | 0.287 |
|  | % | 1.0% | 99.0% | 0.6% | 99.4% | 1.4% | 98.6% |  |  |
| Table | *n* | 494 | 452 | 168 | 295 | 326 | 157 | 155.936 | < 0.001 |
|  | % | 53.6% | 46.4% | 33.2% | 66.8% | 73.5% | 26.5% |  |  |
| Cupboard/chest of drawers | *n* | 9 | 937 | 3 | 460 | 6 | 477 | 3.854 | 0.018 |
|  | % | 0.9% | 99.1% | 0.3% | 99.7% | 1.5% | 98.5% |  |  |
| Cooking source | *n* | 40 | 908 | 8 | 455 | 32 | 451 | 12.055 | 0.003 |
|  | % | 4.1% | 95.9% | 1.8% | 98.2% | 6.4% | 93.7% |  |  |
| Water source | *n* | 432 | 516 | 168 | 295 | 264 | 219 | 3.393 | 0.144 |
|  | % | 44.7% | 55.3% | 41.8% | 58.2% | 47.5% | 52.5% |  |  |
| Shoes/sandals | *n* | 577 | 369 | 260 | 203 | 317 | 166 | 4.633 | 0.094 |
|  | % | 58.1% | 41.9% | 54.8% | 45.2% | 61.3% | 38.7% |  |  |
| Overcrowding | *n* | 545 | 401 | 219 | 244 | 326 | 157 | 70.214 | < 0.001 |
|  | % | 55.3% | 44.7% | 41.7% | 58.3% | 68.6% | 31.4% |  |  |
| Lighting | *n* | 28 | 918 | 8 | 455 | 20 | 463 | 1.646 | 0.292 |
|  | % | 2.6% | 97.4% | 1.9% | 98.1% | 3.3% | 96.7% |  |  |
| Improved construction materials | *n* | 49 | 897 | 5 | 458 | 44 | 439 | 22.201 | < 0.001 |
|  | % | 4.2% | 95.9% | 1.0% | 99.0% | 7.2% | 92.8% |  |  |
| Toilet facility | *n* | 321 | 625 | 97 | 366 | 224 | 259 | 39.936 | < 0.001 |
|  | % | 34.3% | 65.7% | 24.4% | 75.6% | 44.0% | 56.0% |  |  |
| Improved roofing materials | *n* | 57 | 889 | 11 | 452 | 46 | 437 | 9.567 | 0.011 |
|  | % | 5.2% | 94.8% | 2.9% | 97.1% | 7.5% | 92.5% |  |  |
| Improved flooring materials | *n* | 47 | 899 | 5 | 458 | 42 | 441 | 24.571 | < 0.001 |
|  | % | 4.5% | 95.5% | 1.1% | 98.9% | 7.8% | 92.2% |  |  |
| Motorized vehicle | *n* | 52 | 894 | 13 | 450 | 39 | 444 | 23.770 | < 0.001 |
|  | % | 6.3% | 93.7% | 2.4% | 97.6% | 10.1% | 89.9% |  |  |

*n* number of respondents, *%* weighted percentage

* *χ*2 was calculated using weighted chi-squared tests

Table S3. Household asset ownership in intervention (Aweil South) and control (Aweil West) areas during Wave 2

|  |  | Overall | | Aweil South | | Aweil West | | *χ*²* | *P* |
| --- | --- | --- | --- | --- | --- | --- | --- | --- | --- |
|  |  | N = 907 | | N = 436 | | N = 471 | |  |  |
| Item |  | Yes | No | Yes | No | Yes | No |  |  |
| Electricity | *n* | 17 | 890 | 14 | 422 | 3 | 468 | 0.469 | 0.674 |
|  | % | 2.2% | 97.8% | 2.6% | 97.4% | 1.9% | 98.1% |  |  |
| Television | *n* | 12 | 895 | 5 | 431 | 7 | 464 | 12.081 | 0.006 |
|  | % | 3.7% | 96.3% | 1.3% | 98.7% | 5.7% | 94.3% |  |  |
| Refrigerator | *n* | 5 | 902 | 4 | 432 | 1 | 470 | 2.726 | 0.116 |
|  | % | 0.6% | 99.4% | 1.1% | 98.9% | 0.2% | 99.8% |  |  |
| Wristwatch | *n* | 64 | 843 | 27 | 409 | 37 | 434 | 0.045 | 0.861 |
|  | % | 6.5% | 93.5% | 6.7% | 93.3% | 6.3% | 93.7% |  |  |
| Bed | *n* | 582 | 325 | 195 | 241 | 387 | 84 | 119.068 | < 0.001 |
|  | % | 62.0% | 38.0% | 42.3% | 57.7% | 77.7% | 22.3% |  |  |
| Bank account | *n* | 21 | 886 | 13 | 423 | 8 | 463 | 6.503 | 0.011 |
|  | % | 2.2% | 97.8% | 3.6% | 96.4% | 1.1% | 98.9% |  |  |
| Cupboard/chest of drawers | *n* | 12 | 895 | 8 | 428 | 4 | 467 | 0.432 | 0.664 |
|  | % | 1.6% | 98.4% | 1.9% | 98.1% | 1.3% | 98.7% |  |  |
| Improved construction materials | *n* | 36 | 871 | 15 | 421 | 21 | 450 | 0.026 | 0.904 |
|  | % | 4.3% | 95.7% | 4.2% | 95.8% | 4.4% | 95.6% |  |  |
| Toilet facility | *n* | 282 | 625 | 104 | 332 | 178 | 293 | 18.432 | < 0.001 |
|  | % | 29.8% | 70.2% | 22.5% | 77.5% | 35.6% | 64.4% |  |  |
| Improved roofing materials | *n* | 51 | 856 | 18 | 418 | 33 | 438 | 2.007 | 0.230 |
|  | % | 5.3% | 94.7% | 4.1% | 95.9% | 6.2% | 93.8% |  |  |
| Improved flooring materials | *n* | 34 | 873 | 13 | 423 | 21 | 450 | 1.126 | 0.393 |
|  | % | 3.7% | 96.3% | 2.9% | 97.1% | 4.3% | 95.7% |  |  |
| Fitted door | *n* | 165 | 742 | 54 | 382 | 111 | 360 | 33.039 | < 0.001 |
|  | % | 20.5% | 79.5% | 11.9% | 88.1% | 27.4% | 72.6% |  |  |
| **Bucket** | *n* | 447 | 460 | 186 | 250 | 261 | 210 | 0.735 | 0.476 |
|  | % | 52.5% | 47.5% | 50.9% | 49.1% | 53.8% | 46.2% |  |  |
| **Soap** | *n* | 576 | 331 | 233 | 203 | 343 | 128 | 11.712 | 0.004 |
|  | % | 63.7% | 36.3% | 57.6% | 42.4% | 68.6% | 31.4% |  |  |
| **Pen or pencil** | *n* | 415 | 492 | 145 | 291 | 270 | 201 | 41.591 | < 0.001 |
|  | % | 47.6% | 52.4% | 35.6% | 64.4% | 57.2% | 42.8% |  |  |
| **Blanket** | *n* | 393 | 514 | 180 | 256 | 213 | 258 | 2.434 | 0.192 |
|  | % | 39.7% | 60.3% | 42.6% | 57.4% | 37.5% | 62.5% |  |  |
| **Metal cooking pot** | *n* | 585 | 322 | 265 | 171 | 320 | 151 | 0.050 | 0.850 |
|  | % | 64.3% | 35.7% | 64.7% | 35.3% | 64.0% | 36.0% |  |  |
| **Metal tools** | *n* | 140 | 767 | 38 | 398 | 102 | 369 | 11.165 | 0.002 |
|  | % | 13.3% | 86.7% | 9.1% | 90.9% | 16.7% | 83.3% |  |  |
| Motorized vehicle | *n* | 81 | 826 | 21 | 415 | 60 | 411 | 16.404 | 0.001 |
|  | % | 9.7% | 90.3% | 5.2% | 94.8% | 13.2% | 86.8% |  |  |

*n* number of respondents, % weighted percentage. Simpler items newly introduced into Wave 2 survey were bolded. Metal tools excluded knives or cooking utensils

* *χ*2 was calculated using weighted chi-squared tests

Table S4. Characteristics of SMC-eligible children, caregivers and households in intervention (Aweil South) and control (Aweil West) areas during Wave 1

| **Variable** | | **Category** | **Aweil South**  **(N=463)** | | **Aweil West**  **(N=483)** | | **χ^2*^** | **df** | ***P*** | **Total (N=946)** | |
| --- | --- | --- | --- | --- | --- | --- | --- | --- | --- | --- | --- |
|  |  |  | *n* | % | *n* | % |  |  |  | *n* | % |
| **Child** | **Fever** | Yes | 289 | 58.48 | 410 | 84.01 | 8.3803 | (1,31) | 0.007 | 699 | 71.45 |
|  |  | No | 174 | 41.52 | 73 | 15.99 |  |  |  | 247 | 28.55 |
|  | **RDT-confirmed malaria** | Yes | 218 | 48.04 | 255 | 45.71 | 1.2026 | (1.47, 45.50) | 0.298 | 473 | 46.86 |
|  |  | No | 3 | 0.66 | 13 | 4.25 |  |  |  | 16 | 2.48 |
|  |  | Unknown (no fever/did not test if fever) | 242 | 51.3 | 215 | 50.04 |  |  |  | 457 | 50.66 |
|  | **Sex** | Male | 221 | 46.25 | 229 | 47.46 | 0.1187 | (1,31) | 0.733 | 450 | 46.86 |
|  |  | Female | 242 | 53.75 | 254 | 52.54 |  |  |  | 496 | 53.14 |
|  | **Age (years)** | 3–12 months | 43 | 8.43 | 70 | 8.78 | 0.4838 | (3.14, 97.36) | 0.703 | 113 | 8.61 |
|  |  | 1 | 107 | 21.96 | 102 | 24.57 |  |  |  | 209 | 23.28 |
|  |  | 2 | 83 | 19.71 | 92 | 21.99 |  |  |  | 175 | 20.87 |
|  |  | 3 | 129 | 27.53 | 108 | 20.61 |  |  |  | 237 | 24.01 |
|  |  | 4 | 68 | 13.44 | 81 | 18.21 |  |  |  | 149 | 15.86 |
|  |  | 5 | 33 | 8.94 | 30 | 5.85 |  |  |  | 63 | 7.37 |
| **Caregiver** | **Age (years)** | Under 20 | 89 | 13.86 | 62 | 8.48 | 1.407 | (3.12, 96.72) | 0.118 | 151 | 11.13 |
|  |  | 20–29 | 150 | 30.31 | 195 | 43.66 |  |  |  | 345 | 37.09 |
|  |  | 30–39 | 158 | 40.22 | 141 | 31.86 |  |  |  | 299 | 35.98 |
|  |  | 40–49 | 47 | 10.8 | 66 | 11.76 |  |  |  | 113 | 11.29 |
|  |  | 50 or more above | 19 | 4.81 | 19 | 4.24 |  |  |  | 38 | 4.52 |
|  | **Sex** | Male | 138 | 27.73 | 92 | 15.91 | 5.5232 | (1,31) | 0.025 | 230 | 21.73 |
|  |  | Female | 325 | 72.27 | 391 | 84.09 |  |  |  | 716 | 78.27 |
|  | **Partnership status** | Married/partnered | 432 | 95.34 | 424 | 91.42 | 2.1298 | (1,31) | 0.155 | 856 | 93.35 |
|  |  | Non-partnered | 31 | 4.66 | 59 | 8.58 |  |  |  | 90 | 6.65 |
|  | **Literacy** | Yes | 174 | 39.24 | 211 | 43.18 | 0.1736 | (1,31) | 0.680 | 385 | 41.24 |
|  |  | No | 289 | 60.76 | 272 | 56.82 |  |  |  | 561 | 58.76 |
|  | **Education** | None | 313 | 67.27 | 245 | 47.72 | 3.9671 | (1.34, 41.53) | 0.042 | 558 | 57.34 |
|  |  | Informal/religious | 82 | 18.67 | 68 | 12.36 |  |  |  | 150 | 15.47 |
|  |  | Primary or above | 68 | 14.06 | 170 | 39.92 |  |  |  | 238 | 27.19 |
|  | **Occupation** | Non-employed | 79 | 11.98 | 57 | 15.37 | 1.41 | (1.65, 51.07) | 0.252 | 136 | 13.7 |
|  |  | Unemployed | 13 | 1.91 | 10 | 3.61 |  |  |  | 23 | 2.77 |
|  |  | Agricultural | 352 | 82.7 | 353 | 69.26 |  |  |  | 705 | 75.87 |
|  |  | Unskilled manual work | 4 | 0.57 | 40 | 7.13 |  |  |  | 44 | 3.9 |
|  |  | Skilled/Service/professional | 15 | 2.85 | 23 | 4.62 |  |  |  | 38 | 3.75 |
|  | **Caregiver knowledge of SMC purpose** | Yes | 138 | 84.66 |  |  |  |  |  | 138 | 93.35 |
|  |  | No | 25 | 15.34 |  |  |  |  |  | 25 | 6.65 |
|  | **Caregiver knowledge of SMC eligibility** | Yes | 147 | 92.12 |  |  |  |  |  | 147 | 41.24 |
|  |  | No | 16 | 7.88 |  |  |  |  |  | 16 | 58.76 |
| **Household** | **Net ownership** | Yes | 165 | 30.32 | 339 | 72.28 | 23.3778 | (1,31) | < 0.001 | 504 | 51.63 |
|  |  | No | 298 | 69.68 | 144 | 27.72 |  |  |  | 442 | 48.37 |
|  | **Selected child used mosquito net night before survey** | Yes | 143 | 27.09 | 333 | 70.82 | 26.2954 | (1,31) | < 0.001 | 476 | 50.7 |
|  |  | No | 320 | 72.91 | 150 | 29.18 |  |  |  | 470 | 49.3 |
|  | **Household use of other malaria prevention night before survey** | Yes | 50 | 14.3 | 30 | 5.02 | 1.5973 | (1,31) | 0.216 | 80 | 9.59 |
|  |  | No | 413 | 85.7 | 453 | 94.98 |  |  |  | 866 | 90.41 |

*n* number of respondents, % weighted percentage. *BHW boma* health workers, *SPAQ* sulfadoxine-pyrimethamine and amodiaquine, *RDT* rapid diagnostic test, *SMC* seasonal malaria chemoprevention. Blank cells indicate no intervention in Aweil West (control region), therefore no corresponding survey variables. The absence of a wealth index variable in this table, unlike Table 2 (Wave 2) and Table 3 (Wave 3), is due to the fact that wealth quintiles were not generated during the Wave 1 preliminary survey phase. These quintiles were only developed in Wave 2 and Wave 3 using data from earlier surveys (see Statistical Analysis)

* *χ*2 was calculated using weighted chi-squared test
